# Metal removal capability of two cyanobacterial species in autotrophic and mixotrophic mode of nutrition

**DOI:** 10.1186/s12866-022-02471-8

**Published:** 2022-02-17

**Authors:** Elham Ghorbani, Bahareh Nowruzi, Masoumeh Nezhadali, Azadeh Hekmat

**Affiliations:** 1 Department of Biotechnology, Faculty of Converging Sciences and Technologies, Islamic Azad University, Science and Research Branch, Tehran, Iran; 2 Department of Biology, Islamshahr Branch, Islamic Azad University, Islamshahr, Iran

**Keywords:** Metal removal capability, Extracellular polymeric substances, *Nostoc*, Cyanobacteria, Mixotrophic Media culture

## Abstract

**Background:**

Cyanobacteria are ecologically significant prokaryotes that can be found in heavy metals contaminated environments. As their photosynthetic machinery imposes high demands for metals, homeostasis of these micronutrients has been extensively considered in cyanobacteria. Recently, most studies have been focused on different habitats using microalgae leads to a remarkable reduction of an array of organic and inorganic nutrients, but what takes place in the extracellular environment when cells are exposed to external supplementation with heavy metals remains largely unknown.

**Methods:**

Here, extracellular polymeric substances (EPS) production in strains *Nostoc* sp. N27P72 and *Nostoc* sp. FB71 was isolated from different habitats and thenthe results were compared and reported.

**Result:**

Cultures of both strains, supplemented separately with either glucose, sucrose, lactose, or maltose showed that production of EPS and cell dry weight were boosted by maltose supplementation. The production of EPS (9.1 ± 0.05 μg/ml) and increase in cell dry weight (1.01 ± 0.06 g/l) were comparatively high in *Nostoc* sp. N27P72 which was isolated from lime stones.The cultures were evaluated for their ability to remove Cu (II), Cr (III), and Ni (II) in culture media with and without maltose. The crude EPS showed metal adsorption capacity assuming the order Ni (II) > Cu (II) > Cr (III) from the metal-binding experiments.Nickel was preferentially biosorbed with a maximal uptake of 188.8 ± 0.14 mg (g cell dry wt) ^−1^ crude EPS. We found that using maltose as a carbon source can increase the production of EPS, protein, and carbohydrates content and it could be a significant reason for the high ability of metal absorbance. FT-IR spectroscopy revealed that the treatment with Ni can change the functional groups and glycoside linkages in both strains. Results of Gas Chromatography-Mass Spectrometry (GC–MS) were used to determine the biochemical composition of *Nostoc* sp. N27P72, showed that strong Ni (II) removal capability could be associated with the high silicon containing heterocyclic compound and aromatic diacid compounds content.

**Conclusion:**

The results of this studyindicatede that strains *Nostoc* sp. N27P72 can be a good candidate for the commercial production of EPS and might be utilized in bioremediation field as an alternative to synthetic and abiotic flocculants.

**Supplementary Information:**

The online version contains supplementary material available at 10.1186/s12866-022-02471-8.

## Highlight

Mixotrophic conditions can be used to optimize the biosorption controllable factors for the maximum metal removal efficiency.

Different habitats (lime stones of Khuzestan province and fresh water of of Golestan province) can effect the uptake of heavy metal by two Nostoc species (Nostoc sp. N27P72 and Nostoc sp. FB71).

Modification of culture media can maximize the uptake capacity of the heavy metal ions by two Nostoc species.

**Supplementary Information:**

The online version contains supplementary material available at 10.1186/s12866-022-02471-8.

## Introduction

Cyanobacteria are an extremely diverse groups of prokaryotes whose adaptive capacity along with the ability to tolerate extreme conditions makes them ubiquitous in aquatic [1], and terrestrial environments [2]. They are well known as producers of a wide range of natural compounds which are in turn recognized as toxins that can be potential and useful in pharmaceutical industry [3–10] Genus *Nostoc* is a large and morphologically diverse group of phototrophic cyanobacteria which has found in various habitats. They have drawn more attention because of the presence of outermost polysaccharidic envelopes, often coupled with the capability to release exocellular polysaccharides (RPS) in the culture medium during cell growth [11, 12]. Most of these polymers are characterized by an anionic nature, owing to the presence of uronic acids and/or of other charged groups [13]. As a result, polysaccharides typically have very high affinity to metallic ions and can be considered as a very promising chelating agents for the removal of heavy metals from water [14–18].

Many factors affect cell growth,metabolite accumulation and extracellular polymeric substances (EPSs) including nutrients, such as phosphate and nitrogen, temperature, light intensity, aeration rate, and mixotrophic condition, in microalgal cultures [19, 20] Although the presence of EPSs are extremely preserved among cyanobacteria, there is not much information about factors that maximize thebiosynthesis of EPSs and affects the biosorption capacity [15, 21–24]. Several trace elements such as copper, cobalt, and nickel are essential cofactors in cyanobacteria strains. Unlike organic contaminants, heavy metals such as copper and lead are the main pollutants of freshwater due to persistent, toxicity, recalcitrant, and non-biodegradable nature [25]. On the other hand, heavy metal ions concentration at low concentrations are known to be toxic to the organisms because they inhibit many enzymes irreversibly. Heavy metal uptake capacity of algal biomass has proved to be the highest due to the presence of polymers containing functional groups (which can act as binding sites for metals) such as amino, hydroxyl, carboxyl, and sulfate.Polysaccharides, proteins, or lipids on the cell wall surface which are good examples for these polymers [11] Several studies have been conducted on phytoremediation investigation and several authors have established the fact that treatment of wastewaters using algae, and particularly microalgae decrease organic and inorganic nutrients, such as toxic chemicals remarkably [26–30]. Our previous study indicated that cell growth and the production of EPSs are highly culture conditions dependent. There is no correlation between cell growth and the production of EPSs in cultures with different sources of nitrogen. In contrast, light intensity and cell growth in mixotrophic conditions have had a highly positive effect on the production of EPSs. In salt-grown cultures, thick layers of ASN_M strain supports the cells from NaCl stress hence its growth is maintained without the NaCl stress inhibition [31].

This study evaluates the metal removal capability of two *Nostoc* species isolated from different habitats in autotrophic and mixotrophic media cultures. We hypothesis that the strain of *Nostoc* sp. N27P72 that is isolated from lime stones of Khuzestan province have many features includinghigh tolerance to different abiotic stress such as drought, and high light intensity that make it an ideal candidate for the selective removal and concentration of heavy metals, compared to aquatic strain.

The main aim of this study is utilizing the mixotrophic conditions to optimize the biosorption controllable factors to achieve the maximum heavy metal removal efficiency of algal biomass. Lead, Nickel and copper were selected because of their contrasting toxicity and essentiality. Furthermore, cell dry weight, carbohydrates content, total soluble proteins, analysis of functional groups have been and Chemical composition of the lyophilized EPSs has been investigated.

## Materials and methods

### Materials

All materials and reagents were purchased from Sigma-Aldrich unless otherwise specified.

### Cyanobacterial strains

*Nostoc* sp. N27P72 and *Nostoc* sp. FB71 were obtained from the Cyanobacteria Culture Collection (CCC) and the ALBORZ Herbarium, at the Science and Research Branch, Islamic Azad University, Tehran. Originally *Nostoc* sp. N27P72 and *Nostoc* sp. FB71 was obtained from the lime stones of Khuzestan province and fresh water of Golestan province respectively.

### Culture conditions

*Nostoc* sp. N27P72 was cultered on modified Z8IX medium and *Nostoc* sp. FB71 was cultured on liquid media BG11 medium (nitrate free) [32] (Rippka et al. 1979) and the pH was adjusted to 7.1.

the cultures were used to optimize the metal removal capability of the broth (25 ml in 250 ml baffled shake flasks) containing the sugars glucose, maltose, lactose, and sucrose, separately, as additional carbon sources at the concentrations of 10 g/l. *Nostoc* sp. N27P72 and *Nostoc* sp. FB71 were cultured for 48 h.

Cultures were incubated in a culture chamber at 28 °C and were provided with continuous artificial illumination of approximately 15 μmol m^−2^ s^−1^ for two weeks [33].

### Determination of cell biomass

The cells were harvested and dried in an oven set at 100 °C. The cell dry weight was measured after 6, 12, 24, and 48 h [31, 34].

### Isolation of exopolysaccharides

Strains *Nostoc* sp. N27P72 and *Nostoc* sp. FB71 were harvested after 6, 12, 24, and 48 h from the culture, then they were centrifuged at 1792 g for 30 min at 4 °C (SigmaPK). The EPSs were precipitated by adding ethanol and storing overnight at 4 °C. Precipitates were harvested and put in a fume hood to evaporate the remaining ethanol. Finally, the precipitates were dissolved in milliQ water and lyophilized (Labconco freeze dry system) to obtain the crude EPSs [31].

### Selectivity in the heavy metal removal

The cyanobacterial species were cultured in Z8IX and BG11 media culture containing maltose and without maltose as control culture, then they were tested for their ability to remove Cu (II), Cr (III), and Ni (II). The cultures (400 ml of cell suspensions in 1000 ml Erlenmeyer flasks) were cultured for 10–15 days in an orbital Incubator (Gallenkamp, Loughborough, UK) at 30 ± 1^0^C under continuous illumination provided by cool white fluorescent tubes giving a mean photon flux of 100 μmol photons m^−2^ s^−1^ photosynthetic active radiation at the flask surface. Before their use for the experiments, aliquots of the cultures were confined in dialysis tubing and pretreated with 0.1 N HCl and then dialyzed against water. Next, the cultures were suspended into metal solutions with continuous stirring. Working solutions of 10 mg l^−1^ Cr (III), Cu (II), and Ni (II) were prepared, (using dilution of 1000 mg l^−1^ standard solutions (pH 5.0) for each metal). the experiments were performed in a thermostat at 25 ± 1 °C. For the determination of the kinetics of metal removal, 5 ml samples were withdrawn five-time every 30 min (0 to 90 min), centrifuged (10 min at 10 000 g), and filtered through a 0.45 μm membrane. The metal uptake was calculated via evaluatimg the difference between the concentrations of the metals in solution byan Atomic adsorption spectrometer (SpectrAA 10 plus, Varian, CA, USA), at the beginning and end of exposure with the cyanobacterial cell suspensions; Cu(II), Ni(II), and Cr(III) concentrations were determined at 232.0, 359.9 and 324.7 nm respectively. All the experiments were performed at least in triplicate, and the data was reported as mean values ± standard error of the mean. The metal uptake q, expressed as mg of metal removed per g of dry biomass, was calculated as q = (C_i_—C_t)_ / m. The concentration of the biomass was determined as dry weight (g l^−1^) by filtering the cell suspension on 0.45 lm filters and by drying the filters at 100 °C until constant weight [35, 36].

To confirm the affection of the metals and determine the interaction of the cations within the different functional groups of the EPSs, metal solution and all incubated EPSs were lyophilized after 24 h dialysis (lyophilized EPSs containing maltose and metal solution of Cu (II), Cr (III) and Ni (II). Total produced EPSs, total soluble proteins, carbohydrate content, and chemical composition were measured.

### Analyzing of carbohydrates content

5 mg of the specific sample culture of each cyanobacterial species were mixed with 2.5 cm^3^ of Antrone reagent (0.65%) in H_2_SO_4_ (65%). The mixture was incubated for 35 min at 100 °C. The absorbance was measured at 620 nm. Carbohydrates content was computed using a calibration curve [37].

## Estimation of total soluble proteins

20 mg of EPS were resuspended in 1 ml of deionized water and sonicated and successively diluted until a homogenous solution was obtained. Protein content was determined according to Lowry et al. (1951), and a calibration curve was constructed using serum albumin.

### Analysis of functional groups by FT-IR

2 mg of lyophilized EPSs was grinded with 100 mg dry KBr and pressed into a mold in a uniaxial hydraulic press. FT-IR spectra of the purified EPSs fractions were recorded in the 4000–400 cm^−1^ region using a FT-IR system (Nicolet is5, Ther-moFisher Scientific). The determinations were performed in two independent replicates and were reported as the mean with a standard error of the mean [38].

### Chemical composition of the lyophilized EPS

Chemical composition of extracts of *Nostoc* sp. N27P72 was evaluated by a coupled gas chromatography –mass spectrometry (GC–MS). The separation of compounds and their analysis was performed by agilent 7000 series Quadrupole GC–MS system with electron impact ionization. The total GC run time was 32 min and the carrier gas was helium. The initial oven temperature was held at 90 °C for 1 min and then reached 300 °C in 13 min, then it was held at this temperature for 20 min. the injector temperature was 300 °C. Interpretation on mass spectrum GC–MS was conducted using the database of National Institute Standard and Technology (NIST) having more than 62,000 patterns and Fiehn Mass Spectra Libraries. The spectrum of the unspecified component was equaled with the spectrum of the identified components stored in the NIST library. The Name, Molecular weight, and Structure of the components of the test materials were ascertained [39].

### Statistical analysis

Results of each representative experiment were analyzed by ANOVA, using the statistical software package SPSS version 24. A significance level of 95% was considered to indicate a statistical difference. The Tukey test was performed to evaluate the significance of difference among mean values when a significant variation (*p* < 0.05) was found by the ANOVA test. Each treatment underwent three replicated measurements for which the mean values ± standard error of mean were obtained [31].

## Results

### Growth and EPS production

**Fig. 1:**
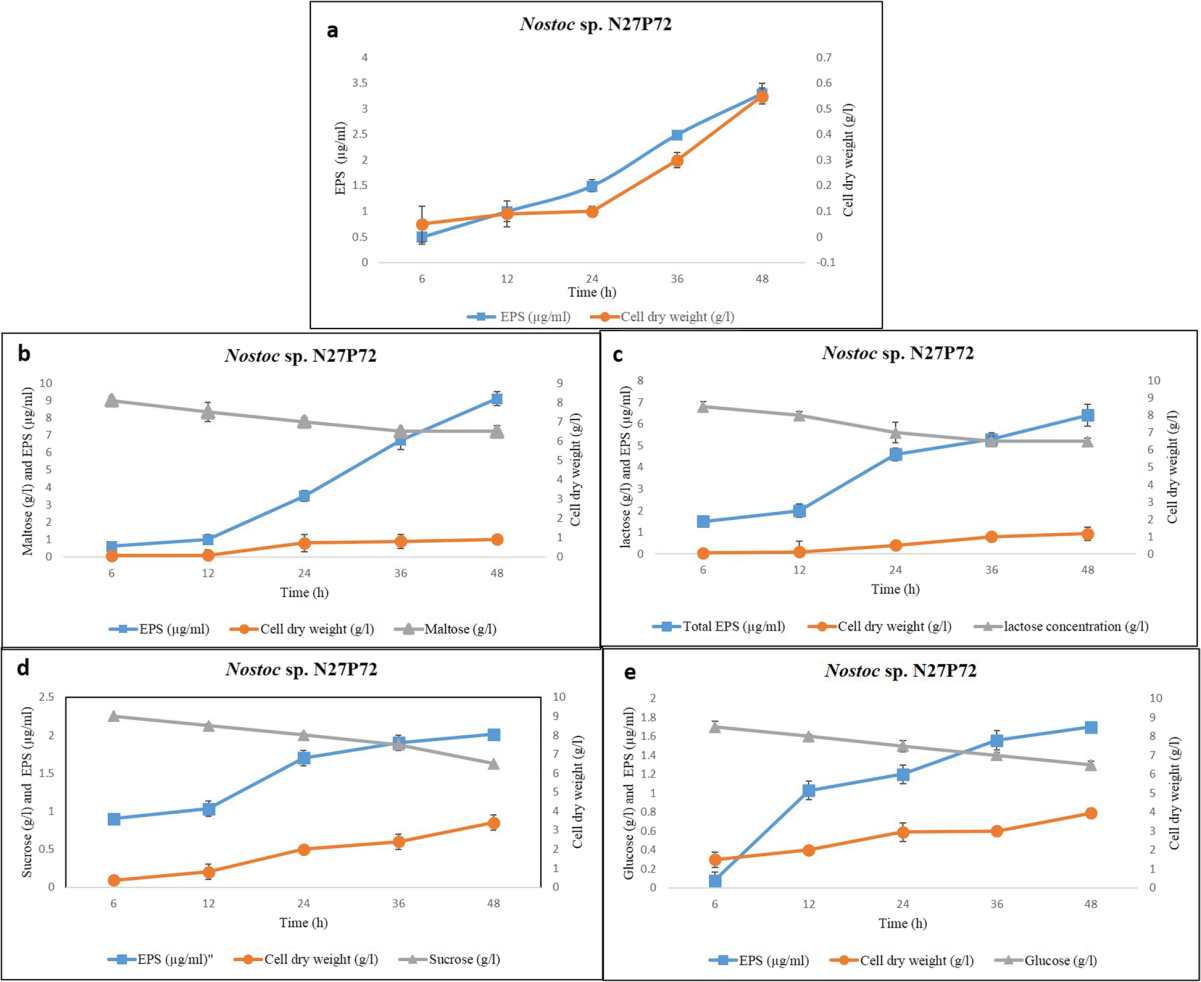
Growth profile and the production of EPSs of *Nostoc* sp. N27P72 cultivated in media culture without additional sugars as a control **a** and media culture containing (10 g/l) maltose **b**, lactose **c**, sucrose **d** and glucose **e** separately. Symbols indicate (●) for cell dry weight (g/l), (□) for total EPS concentration (μg/ml) and (∆) for sugar concentration (g/l) in the media. Results are the mean of duplicated measurement

**Fig. 2:**
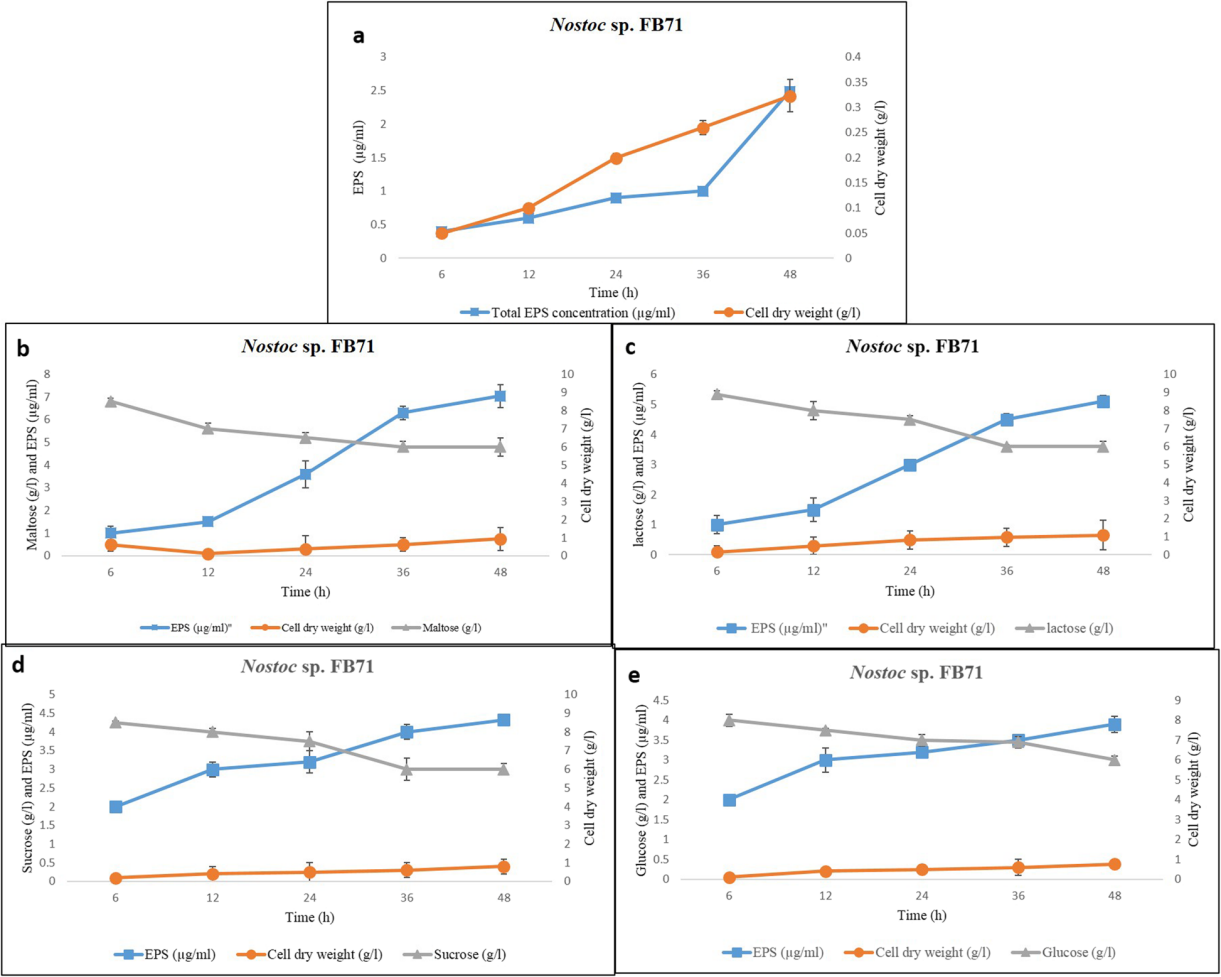
Growth profile and the production of EPSs of *Nostoc* sp. FB71 cultivated in media culture without additional sugars as a control **a** and media culture containing (10 g/l) maltose **b**, lactose **c**, sucrose **d** and glucose **e**. Symbols indicate (●) for cell dry weight (g/l), (□) for total EPS concentration (μg/ml) and (∆) for sugar concentration (g/l) in the media. Results are the mean of duplicated measurement

The two *Nostoc* strains exhibited a distinct difference in growth behavior. As initial trials with,both strains showed that use of disaccharides (maltose, lactose, and sucrose) as carbon source supplementation generally resulted in higher production of EPSs than the use of monosaccharides (Glucose), moreover the amounts of the total produced EPSs, cell dry weight, and residual sugar content were higher in *Nostoc* sp. N27P72 in comparison to *Nostoc* sp. FB71 in all treatments. Consumption of the monosaccharide glucose and the disaccharides lactose and maltose were verified (Fig. 1, 2 and Fig. S1). In all situations, the production of EPSs was started in the exponential growth phase and was shown to remain in the stationary phase. The consumption rate of glucose, maltose, lactose, and sucrose was nearly uniform in both strains and after 48 h all glucose was not consumed and the residual concentration was 6–6.5 ± 0.1 g/l in all treatments using one way ANOVA (*p* < 0.05). The change in 'cell dry weight was very obvious in both strains in cultures adding maltose and reaching 1.01 ± 0.06 g/l in *Nostoc* sp. N27P72 and 0.75 ± 0.16 g/l in *Nostoc* sp. FB71 at 48 h (Fig. 1, 2 and Fig. S1). The increase in cell dry weight might be due to the consumption of produced EPSs, which was reaching a final EPSs concentration of 9.1 ± 0.05 μg/ml in *Nostoc* sp. N27P72 and 7.04 ± 0.1 μg/ml in *Nostoc* sp. FB71 (at 48 h). The experiments using cultures without added sugar showed a slightly lower maximum cell mass (0.55 ± 0.03 g/l in *Nostoc* sp. N27P72 and 0.323 ± 0.05 in *Nostoc* sp. FB71 after 48 h) and the final EPS concentration was 3.3 μg/ml in *Nostoc* sp. N27P72 and 2.5 μg/ml in *Nostoc* sp. FB71 after 48 h. This indicates that the 10 g/L maltose addition had a small boosting effect on both cell mass and EPS production.

The addition of glucose did not stimulate cell growth and EPSs production in *Nostoc* sp. N27P72, however, it was stimulating in EPS production in *Nostoc* sp. FB71. The consumption rate of glucose during the first 6 h was 1.5 g/l in *Nostoc* sp. N27P72 and 2 g/l in *Nostoc* sp. FB71 but decreased to 0. 5 g/l h (6–15 h) and reaching a cell mass of 0.59 ± 0.21 g/l in *Nostoc* sp. N27P72 and 0.25 g/l in *Nostoc* sp. FB71 at 24 h. After 48 h the cell mass finally reached 0.79 ± 0.35 g/l in *Nostoc* sp. N27P72 and 0.38 g/l in *Nostoc* sp. FB71, while the production of EPSs continued (1.7 μg/ml in *Nostoc* sp. N27P72 and 3.9 μg/ml) during the whole 48 h of cultivation. The cell mass obtained in maltose supplemented cultivations resembled that of the lactose supplementation (0.4 ± 0.28 g/l in *Nostoc* sp. N27P72 and 0.5 ± 0.1 g/l in *Nostoc* sp. FB71 after 24 h, maintained at 24 h as 0.94 ± 0.14 g/l in *Nostoc* sp. N27P72 and 0.66 ± 0.09 g/l in *Nostoc* sp. FB71. The EPSs concentrationreached to 6.41 ± 0.13 μg/ml in *Nostoc* sp. N27P72 and 5.10 ± 0.26 μg/ml in *Nostoc* sp. FB71 (after 48 h). Sucrose supplemented cultivations showed that the maximum cell concentration was 1.15 ± 0.21 g/l and the concentration of produced EPSs was 2.01 ± 0.15 μg/ml in *Nostoc* sp. N27P72 and 4.32 ± 0.19 μg/ml in *Nostoc* sp. FB71 after 48 h. The results displayed that while the effects on cell mass were slightly small, production of EPSs amplified upon adding disaccharides such as maltose and lactose at stationary and exponential phases.

### Optimization of metal removal capability

The time course of specific metal removal (q), expressed as mg of metal removed per g of biomass dry weight, by *Nostoc* sp. N27P72 and *Nostoc* sp. FB71 cultivated in media culture containing (10 g/l) maltose, copper, chromium, and nickel in single-metal solutions. The results showed the kinetics of sorption were always rapid for all the metals tested by the cyanobacterial cultures; the saturation of the metal removal capacity of each strain was achieved within the first 10 min in the metal solution.

**Fig. 3:**
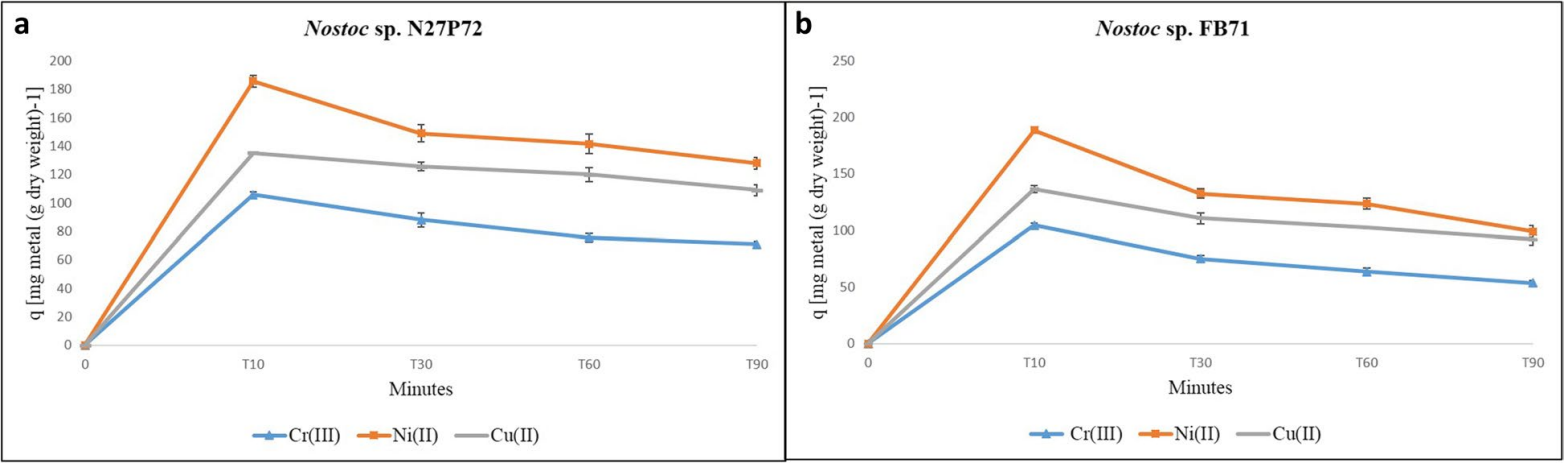
Time course of specific metal removal (q), expressed as mg of metal removed per g of biomass dry weight, by *Nostoc* sp. N27P72 **a** and *Nostoc* sp. FB71 **b** cultivated in media culture containing (10 g/l) maltose, with copper, chromium, and nickel in single-metal solutions. Symbols represent the mean of at least three replicates and bars represent the standard error of the mean, if larger than the dimensions of the symbols, using one-way ANOVA (*p* < 0.05). Symbols indicate () for nickel, (-) for copper, and (▲) for chromium in the media

The metal affinity of the two *Nostoc* strains generally decreased Ni > Cu > Cr respectively. The specific metal uptake (q) was very high, in particular for Ni (Fig. 3 and Fig. S2), which generally were removed in larger amounts compared to Cu and Cr. Among the strains tested, the highest q values towards Ni was 188.8 ± 0.14 mg Ni (g cell dry wt)^−1^ shown by *Nostoc* sp. N27P72, while the highest values of uptake was 185.5 ± 0.24 by *Nostoc* sp. FB71. In single ion solutions, both *Nostoc* strains tested showed the lowest affinity for Cr namely 105.65 ± 0.34 104.5 ± 0.1 mg metal for *Nostoc* sp. N27P72 and *Nostoc* sp. FB71 (g cell dry wt)^−1^ respectively.

Results of metal removal capability optimization showed that adding maltose in culture media resulted in higher EPSs production, protein, and carbohydrates content in comparison to control in both strains Moreover, the amounts of the total produced EPSs, protein, and carbohydrates content were significantly higher in *Nostoc* sp. N27P72 in comparison to *Nostoc* sp. FB71 using one-way ANOVA (*p* < 0.05). Exopolysaccharides concentration in medium containing maltose and metal solution of Ni (II) was 2.87 and 2.57 μg/ml, while it was 1.72 and 1.8 μg/ml in control for *Nostoc* sp. N27P72 and *Nostoc* sp. FB71 respectively. Protein content in medium containing maltose and Ni (II) was 0.2173 and 0.1814 mg/ml, while it was 0.0797 and 0.0611 mg/ml in control for *Nostoc* sp. N27P72 and *Nostoc* sp. FB71 respectively.

**Fig. 4:**
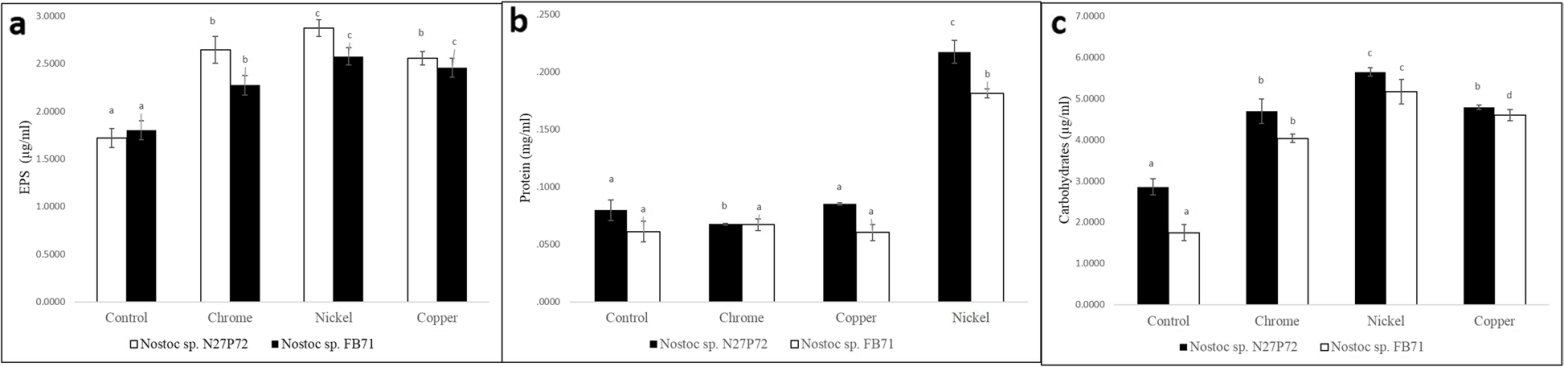
Comparison of the total produced EPSs (μg/ml) **a**, Protein (mg/ml) **b** and Carbohydrates (μg/ml) **c** of *Nostoc* sp. N27P72 and *Nostoc* sp. FB71 in lyophilized EPSs containing maltose and metal solution of Cu (II), Cr (III), and Ni (II). The data present three independent experiments using triplicate samples and mean ± SE values were expressed. The same letters of each line graph are not significantly different from each other, using one-way ANOVA (*p* < 0.05). Symbols indicate () for *Nostoc* sp. N27P72 and () for *Nostoc* sp. FB71

Carbohydrates content in medium containing maltose and Ni (II) was 5.64 and 5.16 μg/ml, while it was 2.85 and 1.74 μg/ml in control for *Nostoc* sp. N27P72 and *Nostoc* sp. FB71 respectively. Nickel removal rate was significantly higher in both strains, which means this metal is more absorbed by polysaccharide envelopes. The reason for removing more nickel (based on the results of the diagrams) is the higher amount of EPSs, proteins, and carbohydrates content compared to other elements. More EPSs, proteins, and carbohydrates content can effectively sequester dissolved metal ions from dilute aqueous solutions (Fig. 4 and Fig. S3).

### Analysis of functional groups

**Fig. 5:**
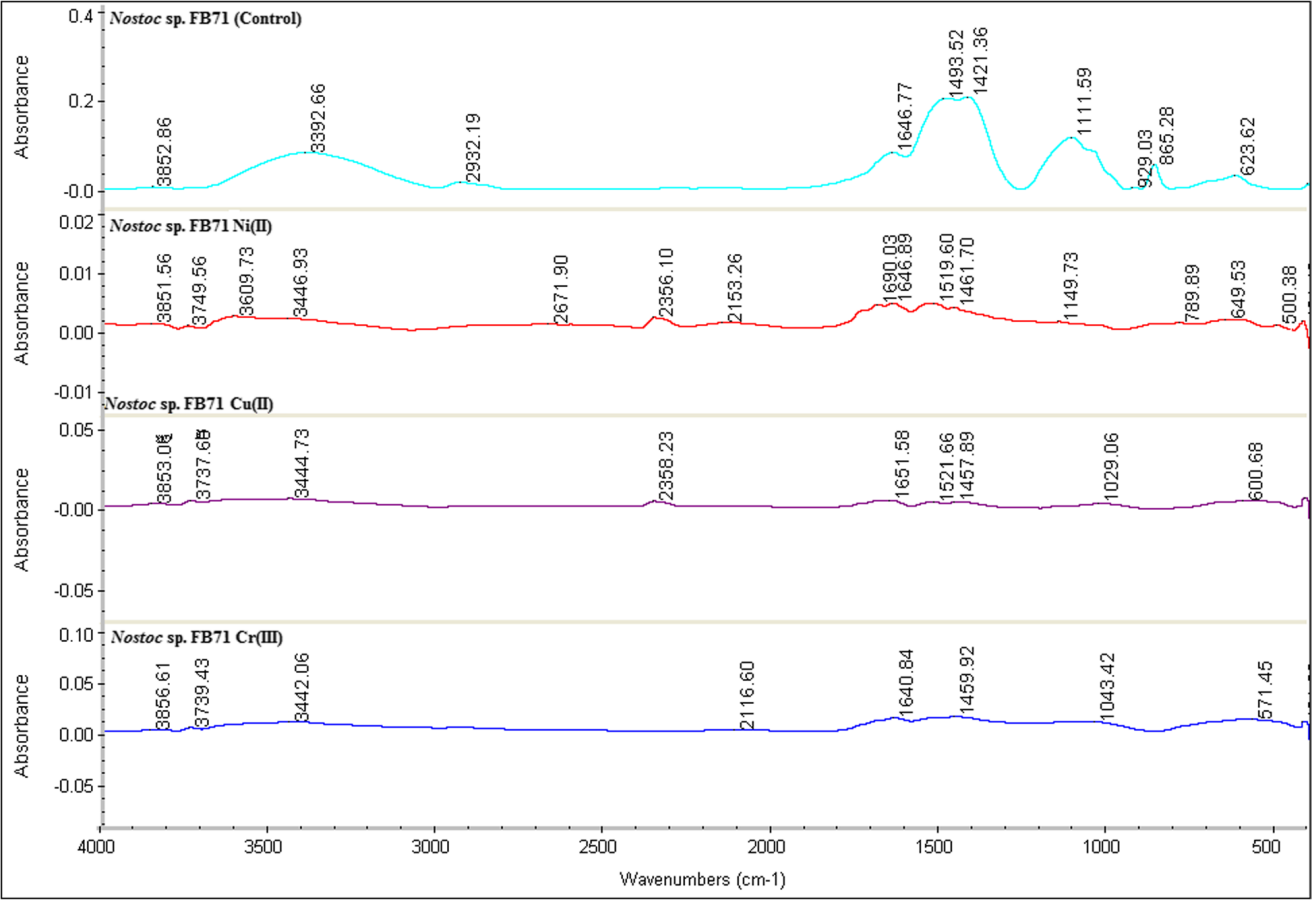
Fourier transforms infrared (FTIR) spectra of EPSs from *Nostoc* sp. FsssB71 against control or exposure to a solution containing 400 ml of cell suspensions in 1000 ml Erlenmeyer flasks of Cu (II), Cr (III), and Ni (II)

**Fig. 6:**
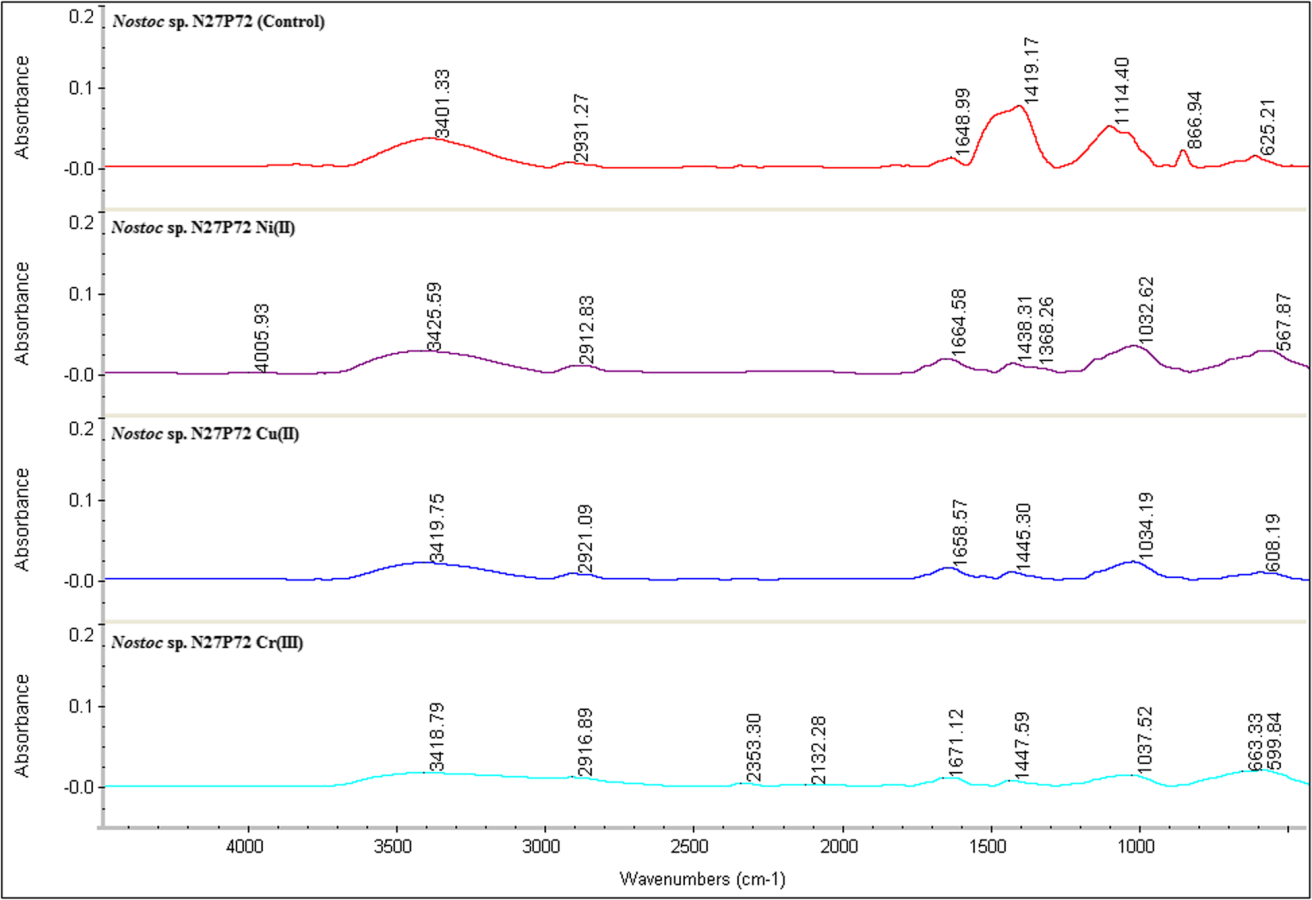
Fourier transforms infrared (FTIR) spectra of EPSs from *Nostoc* sp. N27P72 v.s control or exposure to a solution containing 400 ml of cell suspensions in 1000 ml Erlenmeyer flasks of Cu (II), Cr (III), and Ni (II)

Fourier Transformed Infrared (FT-IR) spectroscopy provided useful information about active functional groups that can be used in the determination of polysaccharide composition. So, the FT-IR spectra was used to evaluate both *Nostoc* sp. N27P72 and *Nostoc* sp. FB71 cultivated in media culture containing (10 g/l) maltose, with copper, chromium, and nickel in single-metal solutions were examined. Strong stretching vibrations for OH and weak stretching vibrations for NH_4_^+^ at 3800 cm^−1^ was detected only in *Nostoc* sp. FB71. The stretching vibration for NH was identified around 3779 cm^−1^ only in *Nostoc* sp. FB71. In both samples stretching vibration for OH was observed at 3400–3440 cm^−1^. The non-asymmetric and asymmetric stretching vibrations for CH was identified at 2854 and 2924 cm^−1^ only in *Nostoc* sp. N27P72. The COOH stretching band (1600–1700 cm^−1^) was observed in both samples and didn’t change after heavy metal treatment. The C-H vibration was observed at 1380–1400 cm^−1^ and a peak at 1384 cm^−1^ corresponding to bending vibration of CH_2_ was only observe at *Nostoc* sp. N27P72 in presence of nickel. A peak at about 1000 cm^−1^ region was associated with C-O polysaccharide and was not observed at *Nostoc* sp. FB71 in presence of nickel. Furthermore, this peak shifted to lower wavenumber at *Nostoc* sp. N27P72 thatcould be related to polysaccharide conformational changes in both strains. Peaks at 1040 and 1029 cm^−1^ were correlated to polysaccharides skeletal and C–O–C and C-O groups of the anomeric region. Aliphatic esters can be observed at 1103 cm^−1^ and this peak was removed due to the interaction of Ni, Cr, and Cu to both strains. The 800–900 cm^−1^ region depicts several vibrational modes corresponding to the type of glycosidic linkages which were removed after treatments of Cr and Cu with both strains. Peaks in 840–860 cm^−1^ region corresponding to α-glucan and 890–910 cm^−1^ corresponding to α and β glycosidase and these peaks were removed in all samples except Ni treatment at *Nostoc* sp. FB71. Peak at 600 cm^−1^ was related to The C-N stretching band (600 cm^−1^) which was detected in both samples but shifted to lower wavenumbers in both strains. Collectively, after treatment of both strains with Ni, Cu, and Cr, the FT-IR patterns for both strains changed obviously (Figs. 5 and 6).

### Chemical composition of the lyophilized EPS by GC–MS

The GC–MS analysis showed cyanobacteria in the presence of heavy metals change aliphatic compound (2-Ethoxyethanol, 3,3-dimethylhexane, Undecane, Dodecane, 2,6,10-trimethyl ، Hydroxylamine, O-decyl ،Tetradecane، Nonadecane، Nonadecane، Propionic acid، Dotriacontane، Eicosane، 2-Methyldecane) and alkanes compounds (Dotriacontane, Dodecane, 2,6,10-trimethyl, 3,3-dimethylhexane, Eicosane) to a rich variety of phytochemical compounds which are effective in heavy metal removal. The active compounds with their retention time (RT), molecular formula, molecular weight, nature of the compound, composition percentage, and quality in the hexane extract are presented in Tables 1, 2, 3 and 4.

**Table 1:** Chemical composition of *Nostoc* sp. N27P72 (control) extracts as revealed by gas chromatography mass spectrophotometry (GC–MS)

Name of Compound	Molecular Formula	Molecular weight	RT (Mins)	Nature of the compound	Area %
2-Ethoxyethanol	C_4_H_10_O_2_	90.12	8.77	hydroxyether	83%
Phenol, 2,4-bis-(1,1-dimethylethyl)	C_17_H_30_OSi	278.5	26.29	Phenolic ester	96%
Dodecane, 2,6,10-trimethyl	C_15_H_32_	212.41	26.41	alkane	64%
3,3-dimethylhexane	C_8_H_18_	114.23	27.15	alkane	40%
Undecane	C_11_H_24_	156.31	28	alkane	78%
Hydroxylamine, O-decyl	C_10_H_23_	173.2957	29.75	alkane	78%
Tetradecane	C_14_H_30_	198.39	29.95	alkane	59%
Nonadecane	C_19_H_40_	268.5	30.20	alkane hydrocarbon	83%
Propionic acid	CH_3_CH_2_CO_2_H	74.08	30.66	Organic acid	35%
Dotriacontane	C_32_H_66_	450.8664	30.68	alkane	64%
Eicosane	C_20_H_42_	282.5	31.69	alkane	83%
2-Methyldecane	C_11_H_24_	156.31	31.99	alkane	53%

**Table 2:** Chemical composition of *Nostoc* sp. N27P72 (Ni(II)) extracts as revealed by gas chromatography mass spectrophotometry (GC–MS)

Name of Compound	Molecular Formula	Molecular weight	Nature of the compound	RT	Area %
pyran	C_5_H_6_O	82.1	six-membered heterocyclic	11.55	49%
Cyclotrisiloxane	H_6_O_3_Si_3_	138.3	Heterocyclic compound	12.02	90%
Cyclotrisiloxane	H_6_O_3_Si_3_	138.3	Heterocyclic compound	15.97	91%
Cyclotrisiloxane	C_5_H_8_O	84.12	Heterocyclic compound	19.62	91%
Cyclotrisiloxane	H_6_O_3_Si_3_	138.3	Heterocyclic compound	23.22	91%
1,2-Benzenedicarboxylic acid	C8H6O4	166.1308	Quinoline Ester	23.51	90%
1,2-Benzenedicarboxylic acid	C8H6O4	166.1308	Quinoline Ester	24.01	91%
1,2-Benzenedicarboxylic acid	C8H6O4	166.1308	Quinoline Ester	24.13	91%
1,2-Benzenedicarboxylic acid	C8H6O4	166.1308	Quinoline Ester	24.35	91%
1,2-Benzenedicarboxylic acid	C8H6O4	166.1308	Quinoline Ester	24.55	91%
1,2-Benzenedicarboxylic acid	C8H6O4	166.1308	Quinoline Ester	24.63	91%
1,2-Benzenedicarboxylic acid	C8H6O4	166.1308	Quinoline Ester	25.04	91%
1,2-Benzenedicarboxylic acid	C8H6O4	166.1308	Quinoline Ester	25.14	91%

**Table 3:** Chemical composition of *Nostoc* sp. N27P72 (Cr(III)) extracts as revealed by gas chromatography mass spectrophotometry (GC–MS)

Name of Compound	Molecular Formula	Nature of the compound	Molecular weight	RT	Area %
Tranylcypromine, pentafluorobenzoyl	C_16_H_10_F_5_NO	ester	327.25	10.5	37%
2-Hexanol	C6H14O	Six carbon alcohol	102.17	10.85	43%
Cyclotrisiloxane	H_6_O_3_Si_3_	Heterocyclic compound	138.3	11.73	64%
Cyclotrisiloxane	H_6_O_3_Si_3_	Heterocyclic compound	138.3	11.83	87%
2H-pyran, 3,4-dihydro-6-methyl	C_6_H_10_O	enol ether	98.14	12.70	60%
2-Butoxyethanol	C_6_H_14_O_2_	glycol ether	118.17	13.35	72%
1,4-pentanediol	C_5_H_12_O_2_	diol	104.15	15.12	40%
Cyclotetrasiloxane	C_23_H_30_O_4_Si_4_	Heterocyclic compound	482.8	15.97	91%
3,5-Hexadien-2-ol	C_6_H_10_O	alcohol	96.13	16.29	9%
6-Bromo-2-hexanone	C_6_H_11_BrO	ketone	179.05	18.40	9%
2-piperidinone	C_5_H_9_NO	lactam	99.13	18.55	9%
Cyclopentasiloxane	H_10_O_5_Si_5_	silicone	230.5	19.63	91%
2,3-dimethylbenzaldehyde	(CH3O)2C6H3CHO	aldehyde	166.17	20.5	20.5
Succinic acid	C_4_H_6_O_4_	Organic acid	118.09	20.64	20.64
Decanal	C_10_H_20_O	aldehyde	156.26	23.04	23.04

**Table 4:** Chemical composition of *Nostoc* sp. N27P72 (Cu(II)) extracts as revealed by gas chromatography mass spectrophotometry (GC–MS)

Name of Compound	Molecular Formula	Molecular	Nature of the compound	RT	Area %
Cyclotrisiloxane	H_6_O_3_Si_3_	138.3	Heterocyclic compound	11.63	78%
Cyclotrisiloxane	H_6_O_3_Si_3_	138.3	Heterocyclic compound	11.71	78%
Cyclotrisiloxane	H_6_O_3_Si_3_	138.3	Heterocyclic compound	15.89	90%
4-penten-2-one	C_5_H_8_O	84.12	methyl ketones	16.31	7%
Cyclotrisiloxane	H_6_O_3_Si_3_	138.3	Heterocyclic compound	19.61	90%
1,2-Benzenedicarboxylic acid	C8H6O4	166.1308	mono ester	23.26	43%
1,2-Benzenedicarboxylic acid	C8H6O4	166.1308	mono ester	23.42	91%
Bis(2-ethylhexyl) phthalate	C_24_H_38_O_4_	390.6	diester of phthalic acid	23.54	80%
1,2-Benzenedicarboxylic acid	C8H6O4	166.1308	mono ester	23.81	91%
1,2-Benzenedicarboxylic acid	C8H6O4	166.1308	mono ester	24.03	91%
1,2-Benzenedicarboxylic acid	C8H6O4	166.1308	mono ester	24.15	91%
1,2-Benzenedicarboxylic acid	C8H6O4	166.1308	mono ester	24.57	91%
1,2-Benzenedicarboxylic acid	C8H6O4	166.1308	mono ester	25.05	91%
1,2-Benzenedicarboxylic acid	C8H6O4	166.1308	mono ester	25.54	91%

**Fig. 7:**
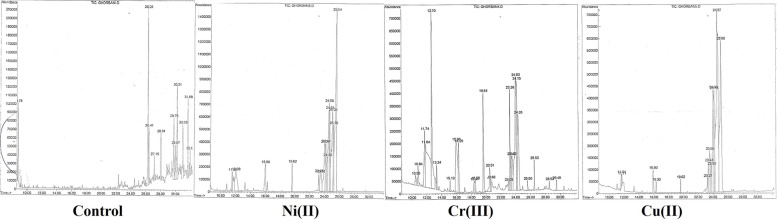
GC–MS chromatogram of the extract of *Nostoc* sp. N27P72, in media culture containing the maltose and without maltose as control culture, were tested for their ability to remove Cu(II), Cr(III), and Ni(II)

The total ion chromatograms (TICs) of all samples demonstrated a strong signal, large peak capacity, and reproducible retention time, indicating the reliability of the metabolomic analysis. However silicon containing heterocyclic compound retention time was not in the same range for the Ni (II) and Cu (II) media culture (11.63 to 23.22), while aromatic diacid compounds retention time for Ni (II) and Cu (II) media culture between 23.26 to 25.14. Moreover, Ni(II) and Cu(II) culture revealed a high composition of cyclic and Ester compounds (Silicon containing heterocyclic compound, Bis(2-ethylhexyl) phthalate and aromatic diacid compounds), while in Cr(III) cultures there were aldehydes compounds *(*Decanal, 2,3-dimethylbenzaldehyde), ketones *compounds (*6-Bromo-2-hexanone), Ester compounds (2-piperidinone), alcoholic compounds (3,5-Hexadien-2-ol, 2-Hexanol, 1,4-pentanediol), ether compounds (2H-pyran, 3,4-dihydro-6-methyl, 2-Butoxyethanol) and cyclic compounds (Silicon containing heterocyclic compound). The strong Ni (II) removal capability of the *Nostoc* sp. N27P72 was attributed to the abundance of Silicon containing heterocyclic compound (91%) and aromatic diacid compounds nds (91%) characterizes by GC–MS (Fig. 7) (Tables 1, 2, 3 and 4).

## Discussion

The possible use of exopolysaccharides producing cyanobacteria for the recovery of valuable metals from industrial wastewater seems to be more promising than most of the other microorganisms [18, 40–42]. The use of cyanobacterial EPSs for biotechnological applications depends on the identification of culture parameters that influence the maximum production of the EPSs [43–45]. Factors such as the amounts of C: N ratio, as well as growth parameters such as light intensity, salinity, and temperature, have been largely disregarded, and very few exhaustive studies on factors influencing the production of cyanobacterial EPSs are available in the liteature [23]. Though, several elements that can stimulate the production of EPSs., especially pH, dilution rate, growth phase, presence/absence of magnesium, calcium, potassium, and heavy metals, as well as the addition of glyoxylate, glucose, citrate, acetate, valerate, and EDTA have been sporadically studied [23]. Moreover, the responses of cyanobacteria to changes in culture conditions appear to be frequently strain-dependent, making the optimization of EPSs production even more difficult [46]. It was suggested that some additives, including amino acids, vitamins, and precursors, may also play an important role in EPSs production during the growth process of cyanobacteria [47].

In this article, we investigated the effect of adding four different sugars to the culture medium (glucose, maltose, lactose, and sucrose) as carbohydrate sources and their effect on the amount of EPSs and cell dry weight in two strains of *Nostoc.* The results of the present investigation indicated that the optimal medium for the production of EPSs by the isolated *Nostoc* sp. N27P72 strain was boosted by maltose supplementation. Previous studies on several bacterial EPSs production reported that amino acids and precursor supplements showed a stimulating effect on bacterial growth and EPSs synthesis, while others demonstrated that neither additional carbohydrates nor amino acids supplementation affects the EPS level [48]. Moreover, among the different nature of carbon sources, carbohydrate sugars are the preferred ones for EPSs production. In this study, maltose was found to be the most efficient carbon source. Nowruzi et al., 2013 showed that light intensity and cell growth in cultures experiencing mixotrophic conditions at either 150 or 50 μmol photon m^–2^ s^–1^ had a strong positive effect on EPSs production [31]. A similar result has been previously reported for other species in this genus [19, 49–51].

The prominent feature of mixotrophic cultures is the presence of two energy sources: organic carbon sources and the light. The former is controlled by the concentration of organic carbon sources, and the latter is influenced by light intensity. This offers the possibility of remarkable increase in the microalgal cell concentration, and hence the EPSs productivity, in batch systems [31].

A study conducted by Fabregas et al. (1999) have shown the ability of *Porphyridium cruentum* to produce 0.33 g·L^−1^ of EPS when grown mixotrophically with 15% potato extract as an organic carbon source, an amount greater compared to the photoautotrophic conditions that were used in the same study [52].

This production yield was lower than the usual amounts of EPSs that were obtained with this strain in optimized photoautotrophic cultures (up to 8.7 g·L^−1^ in the study of Iqbal and Zafar (1993) [53]. For *Chlorella* sp., the three cultivation modes (i.e. photoautotrophic, mixotrophic and heterotrophic) were compared for lipids, exopolymeric substances, and pigments accumulations, showed the highest biomass and EPS concentration (1.14 g·L^−1^) for mixotrophic conditions [54]. The most surprising result was the discovery of the ability of the strain *Neochloris oleoabundans* to produce up to 5 g·L^−1^ of EPS in mixotrophic cultures using lactose as substrate, whereas no production was noticed under photoautotrophy, heterotrophy, or neither mixotrophy using glucose as carbon source [55].

human and industrial activities affected the nature negatively especially via releasing large amounts of toxic elements, especially heavy metals [56] Exopolysaccharides are surface-active mixtures that are suggested to exclude heavy metal pollution. Consequently, microbial EPSs with decent metal adsorption selectivity seem to be precious in extracting valuable metals from industrial wastewater. Bearing this in mind, we studied supplemented media culture as an additional sugar source exopolysaccharides to evaluateits efficiency in adsorbing various toxic heavy metals (Cu, Ni, and Cr) [57]. We found that using maltose as a carbon source was reported to produce a higher amount of EPSs, protein, and carbohydrates content and it could be a reason for the high ability of metal absorbance. This statement has been underlined by Wong and Tam (1984) who stated that algal cells cultivated in the media with very high metal contents also gathered higher metal contents. Despite this, in several samples, the metal uptake was independent of the external metal concentration, this point was coincided with the conclusion of Wetton et al. (1976). *N. muscorum* could grow in wastewater, Interestingly high concentrations of Cu and Mn could not only affect the growth of the microorganism but also promote its growth. This event might happen as a result of the resistance nature of the cyanobacterium to Cu and Mn beside the occurrence of a high content of organic matter, which might detoxify Cu and Mn [58].

The quantity and compactness of different kinds of carbohydrates can help to sort the polysaccharidic layer surrounding the cells which inhibits direct interaction between the cells and toxic heavy metals. recent studies suggested that high viscosity of the cultures, can, delayed the free diffusion of copper ions into the media culture [36]. the presence of negatively charged polysaccharidic layers surrounding cyanobacterial cells such as uronic acids, sulfate, and ketal-linked pyruvate groups may play an important role in the sequestration of metal cations, and informing an environment improved in those metals that are crucial for cell growth but are existing at very low concentrations in some environments [23].

We found that strain of *Nostoc* sp. N27P72 that wasisolated from lime stones of Khuzestan province have many features which include a high amount of EPSs and high tolerance to drought and high density of light that make it an ideal candidate for selective removal and concentration of heavy metals, compared to aquatic strain. Actually, in all of the possible systems in which cyanobacteria were involved, the synthesis of EPSs provides a structurally resistant and hydrated microenvironment, as well as a putative supportive characteristic etoward several risk factors, both chemical and physical. This environment represent a boundary between cells and the immediate outer environment, and supports the cells from toxic heavy metal [59].

The experiments showed that *Nostoc* sp. N27P72 seems to be efficient in metal removal, and its q max (maximum amount of nickel removed per biomass unit) was reported to be 188.8 ± 0.14 mg Ni (g cell dry wt) ^−1^ nickel removed in compare to the value of 185.5 ± 0.24 mg Ni (g cell dry wt) ^−1^ of *Nostoc* sp. FB71 biomass. the metal uptake capacity of EPSs under study (especially for nickel) was remarkable in compare with other biosorbent efficiencies and the lowest affinity seems to be Cr. Our results contradicted the result of Chan et al. (1991) who found the possibility Ni removal from the mixture of 90% electroplating effluent and 10% raw sewage by using two species of *Chlorella* was comparatively low (below 20%) [60]. While Kazy et al. (2002) declared that the production of EPSs was considerably higher in a copper-resistant isolate of *Pseudomonas aeruginosa* compared to its copper-sensitive counterpart [61]. Moreover, Ozturk and Aslim, 2008 found Cr(VI) is an important stress factor that increases EPSs concentration in cyanobacteria [62].

Optimized culture conditions elevate the capitulate of the *Nostoc* sp. N27P72 EPSs, consequently enhance the chances of its commercial-scale production and makes it more acceptable for specific environmental industry applications. However, finding a general pattern for the special effects of metals on EPSs synthesis is very difficult, the effects on EPSs synthesis are metal-specific. In some cases, the shortage of Mg^2+^ and Ca^2+^ elicited the production, whereas there were no effects in other cases. The increase in EPS synthesis appears to develop toxic metals resistance. A study carried out by Ozturk and Aslim 2008, showed that *Chroococcus* and *Synechocystis* strains resistant to Cr (VI) created larger amounts of EPSs compared to the Cr (VI)-sensible isolates [62]. Pereira et al*.* 2013 suggested that there is a promising view that a greater EPSs concentration inspired by the metal, played a role in increasing its immobilization [13]. Though, it was later reported that in *Cyanothece* sp. CCY 0110, the existence of heavy metals expressively affected its protein profile but did not improve the amount of RPS released by the cells [38].

Due to the presence of negatively charged groups, primarily carboxyl groupshave been indicated to have a good sorbent capacity towards positively charged metal ions [63–66]. Among the parameters that strongly affect the metal uptake, the capacity of biopolymers is associated to the metal affinity to their functional groups [67]. Shuhong et. al (2014) and Delattre et.al (2016) reported the implication of O–H, C = O, C–O–C, and C = O-C groups of the EPSs in the binding of Cu^2+^, pb^2+^, and Cr^6+^ ions [67, 68].

GC–MS is an extensively used method for metabolomics researchs, particularly for enabling the identification and quantification of the metabolites involved in the central pathways of primary metabolism such as amino acids, sugar alcohols, sugars, organic acids, and polyamines. In this study, the metabolite profiling analysis has been performed using GC–MS analysis in two *Nostoc* species after 24 h exposure to Cu (II), Cr (III), and Ni (II). The GC–MS analysis of an extract of Ni(II) and Cu(II) *Nostoc* sp. N27P72 revealed cyclic and ester compounds (Silicon containing heterocyclic compound, Bis(2-ethylhexyl) phthalate, and aromatic diacid compounds), compared to the control. These compounds were recorded as absorption agents in the EPSs. In this regard, Agronematol, 2013, investigated the nematicidal potential of different species of cyanobacterial, *Aphanocapsa albida*, *Anabaena oryzae*, *Nostoc muscorum,* and *Calothrix marchica* against the *Meloidogyne incognita* on banana plants. He found that Acetamide, 2-fluoro, Cyclotrisiloxane, hexamethyl, and Oxime-methoxy-phenyl were the major components found in algae by GC–MS. Moreover, Gheda et al., 2020, investigated the natural products from some soil cyanobacterial extracts with potent antimicrobial, antioxidant and cytotoxic activities [69]. They found the presence of Cyclotrisiloxane in phenol compounds of soil cyanobacteria of *Nostoc* by GC–MS. Additionally, Kanimozhi et al., 2021, found the presence of Cyclotrisiloxane in *Microcystis* sp. with synergistic activity against the bacterial pathogens using GC–MS [70].

Wu et al. (2006) attributed the cytotoxicity effects of fatty acids to their ability to increase the membrane permeability leading to membrane damage [71]. Mundt et al. 2003 [72], suggested that fatty acids is produced by cyanobacteria as a defense mechanism against other microorganisms might be able to change the permeability of the cell membrane through interacting with proteins and lipids of the membrane, inhibiting special enzymes by a layer around the cells. Presence compounds of Benzene derivatives in Ni and Cu cultures may be related to absorption compounds to remove the metal.

. it has been previously shown that some benzene inhibited b-ketoacyl-acyl carrier protein synthase III, a condensing enzyme that initiates fatty acid biosynthesis in most cyanobacteria, leading to absorption activity of metal. Furthermore, the cytotoxic activity of the pure compound 1, 2- benzene dicarboxylic acid, mono 2- ethylhexyl ester (DMEHE) from marine-derived actinomycete *Streptomyces* sp. VITSJK8 was examined against mouse embryonic fibroblast (NIH 3T3) and human keratinocyte (HaCaT) normal cell lines, human hepatocellular liver carcinoma (HepG 2), and human breast adenocarcinoma (MCF-7) cell lines by using MTT assay (Krishnan et al., 2014). 1,2-Benzenedicarboxylic acid, bis(2-methyl propyl) ester was recorded by Alghamdi et al., 2018 [73] as a plasticizer and has light and heat stability. The antibacterial properties of olive leaves extract is probablyassociated with the high cyclotrisiloxane hexamethyl content, which has been tested by Keskin e al., 2012 [74]. lastly, as compared to solid wastes created from the old approach used for the heavy metal removal, polysaccharides are natural, nontoxic, and biodegradable polymers, thus reducing their polluting effects and making them attractive for potential use as metal-absorbent safe materials.

## Conclusion

Base on this study, EPSs play a crucial role in protecting the environment from harmful toxic heavy metals. However, despite large number of studies claimed this role, only a few of them directly investigated the modification of metal removal capability by cyanobacteria under mixotrophic conditions. In this study, the highest EPSs production efficiency was found in cultures supplemented by maltose and biomass of *Nostoc* sp. N27P72 possesses a high affinity and a high specific uptake for nickel, comparable with the best performances reported by other microbial biomass, and suggest the possibility to use *Nostoc* sp. N27P72 for the bioremoval of heavy metals from polluted water. The FT-IR spectra showed that treatment of Ni with both strains made obvious changes in functional groups of polysaccharides and linkages. Silicon containing heterocyclic compound and aromatic diacid compounds, a major constituent of *Nostoc* sp. N27P72. in this study, metal removal capability of *Nostoc* sp. N27P72 extract was probably associated with the high Silicon containing heterocyclic compound and aromatic diacid compounds content. Although the key elements controlling the cyanobacterial EPSs production have been recognized, inclusive strain-specific studies taking into account the interaction between the variables to know the system reacts to changes, are still missing. This needs a better fact of the genes and metabolic paths complicated in the mechanism of EPS production in cyanobacteria. In conclusion, the strain *Nostoc* sp. N27P72 may be a suitable candidate for mass production of an ecologically attractive EPSs with a potential use in the bioremediation field.

## Supplementary Information

**Additional file 1:** **Supplementary Fig. S1.**

**Additional file 2:** **Supplementary Fig. S2.**

**Additional file 3:** **Supplementary Fig. S3.**

## Data Availability

All data generated or analysed during this study are included in this published article (Tables 1, 2, 3 and 4 and supplementary information files: S1, S2 and S3). The mass spectrometry spectra data during the current study are available in the ProteomeXchange repository, ( http://www.ebi.ac.uk/pride/archive/projects/PXD030537) and ProteomeXchange accession is PXD030537. **Code availability** Not applicable.

## References

[1] Kumar A, Singh JS. Cyanoremediation: a green-clean tool for decontamination of synthetic pesticides from agro-and aquatic ecosystems. Agro-environmental sustainability. 2017;1:59–83.

[2] Tamaru Y, Takani Y, Yoshida T, Sakamoto T (2005). Crucial role of extracellular polysaccharides in desiccation and freezing tolerance in the terrestrial cyanobacterium Nostoc commune. Appl Environ Microbiol.

[3] Nowruzi B, Lorenzi AS (2021). Production of the neurotoxin homoanatoxin-a and detection of a biosynthetic gene cluster sequence (anaC) from an Iranian isolate of Anabaena. S Afr J Bot.

[4] Nowruzi B, Porzani SJ (2021). Toxic compounds produced by cyanobacteria belonging to several species of the order Nostocales: A review. J Appl Toxicol.

[5] Rajabpour N, Nowruzi B, Ghobeh M (2019). Investigation of the toxicity, antioxidant and antimicrobial activities of some cyanobacterial strains isolated from different habitats. Acta Biologica Slovenica.

[6] Nowruzi B, Khavari-Nejad R-A, Sivonen K, Kazemi B, Najafi F, Nejadsattari T (2012). Identification and toxigenic potential of a Nostoc sp. Algae.

[7] Nowruzi B, Wahlsten M, Jokela J (2019). A report on finding a new peptide aldehyde from cyanobacterium nostoc sp Bahar m by lc-ms and marfey’s analysis. Iran J Biotechnol.

[8] Nowruzi B, Sarvari G, Blanco S. Applications of cyanobacteria in biomedicine. Handbook of Algal Science, Technology and Medicine. Amsterdam: Elsevier; 2020. p. 441-53.

[9] Nowruzi B, Blanco S (2019). In silico identification and evolutionary analysis of candidate genes involved in the biosynthesis methylproline genes in cyanobacteria strains of Iran. Phytochem Lett.

[10] Jafari Porzani S, Konur O, Nowruzi B. Cyanobacterial natural products as sources for antiviral drug discovery against COVID-19. Journal of Biomolecular Structure and Dynamics. 2021;2:1–17. 10.1080/07391102.2021.1899050 33749496

[11] Mota R, Pereira SB, Meazzini M, Fernandes R, Santos A, Evans CA (2015). Effects of heavy metals on Cyanothece sp. CCY 0110 growth, extracellular polymeric substances (EPS) production, ultrastructure and protein profiles. J Proteomics.

[12] Nowruzi B, Haghighat S, Fahimi H, Mohammadi E (2018). Nostoc cyanobacteria species: a new and rich source of novel bioactive compounds with pharmaceutical potential. J Pharm Health Serv Res.

[13] Pereira SB, Mota R, Santos CL, De Philippis R, Tamagnini P (2013). Assembly and export of extracellular polymeric substances (EPS) in cyanobacteria: a phylogenomic approach. Adv Bot Res.

[14] Cepoi L, Zinicovscaia I, Chiriac T, Rudi L, Yushin N, Miscu V (2019). Silver and gold ions recovery from batch systems using Spirulina platensis biomass. Ecol Chem Eng S.

[15] De Philippis R, Faraloni C, Margheri MC, Sili C, Herdman M, Vincenzini M (2000). Morphological and biochemical characterization of the exocellular investments of polysaccharide-producing Nostoc strains from the Pasteur Culture Collection. World J Microbiol Biotechnol.

[16] Suresh Kumar A, Mody K, Jha B (2007). Bacterial exopolysaccharides–a perception. J Basic Microbiol.

[17] Nowruzi B, Bouaïcha N, Metcalf JS, Porzani SJ, Konur O (2021). Plant-cyanobacteria interactions: Beneficial and harmful effects of cyanobacterial bioactive compounds on soil-plant systems and subsequent risk to animal and human health. Phytochemistry.

[18] Ni L, Gu G, Rong S, Hu L, Wang P, Li S (2019). Effects of cyanobacteria decomposition on the remobilization and ecological risk of heavy metals in Taihu Lake. Environ Sci Pollut Res.

[19] Otero A, Vincenzini M (2003). Extracellular polysaccharide synthesis by Nostoc strains as affected by N source and light intensity. J Biotechnol.

[20] Helm RF, Huang Z, Edwards D, Leeson H, Peery W, Potts M (2000). Structural characterization of the released polysaccharide of desiccation-tolerant Nostoc commune DRH-1. J Bacteriol.

[21] Gupta P, Diwan B (2017). Bacterial exopolysaccharide mediated heavy metal removal: a review on biosynthesis, mechanism and remediation strategies. Biotechnology Reports.

[22] Klock J-H, Wieland A, Seifert R, Michaelis W (2007). Extracellular polymeric substances (EPS) from cyanobacterial mats: characterisation and isolation method optimisation. Mar Biol.

[23] Pereira S, Zille A, Micheletti E, Moradas-Ferreira P, De Philippis R, Tamagnini P (2009). Complexity of cyanobacterial exopolysaccharides: composition, structures, inducing factors and putative genes involved in their biosynthesis and assembly. FEMS Microbiol Rev.

[24] Yoshimura H, Kotake T, Aohara T, Tsumuraya Y, Ikeuchi M, Ohmori M (2012). The role of extracellular polysaccharides produced by the terrestrial cyanobacterium Nostoc sp. strain HK-01 in NaCl tolerance. J Appl Phycol.

[25] Micheletti E, Pereira S, Mannelli F, Moradas-Ferreira P, Tamagnini P, De Philippis R (2008). Sheathless mutant of cyanobacterium Gloeothece sp. strain PCC 6909 with increased capacity to remove copper ions from aqueous solutions. Appl Environ microbiol.

[26] Anjana K, Kaushik A, Kiran B, Nisha R (2007). Biosorption of Cr (VI) by immobilized biomass of two indigenous strains of cyanobacteria isolated from metal contaminated soil. J Hazard Mater.

[27] Devi YM, Mehta S (2014). Changes in antioxidative enzymes of cyanobacterium Nostoc muscorum under copper (Cu2+) stress. Sci Vision.

[28] Essa AM, Mostafa SS (2011). Heavy metals biomineralization by some cyanobacterial isolates. Egypt J Bot.

[29] Farooqui A, Suhail S, Zeeshan M (2017). Cadmium induced oxidative stress and biochemical responses in cyanobacterium Nostoc muscorum. Russ J Plant Physiol.

[30] Principe MV, Permigiani IS, Della Vedova MC, Petenatti E, Pacheco P, Gil RA (2020). Bioaccessibility studies of Fe, Cu and Zn from Spirulina dietary supplements with different excipient composition and dosage form. J Pharm Pharmacogn Res.

[31] Nowruzi B, Khavari-Nejad R, Sivonen K, Kazemi B, Najafi F, Nejadsattari T (2013). Optimization of cultivation conditions to maximize extracellular investments of two Nostoc strains. Arch Hydrobiol Suppl Algol Stud.

[32] Rippka R, Deruelles J, Waterbury JB, Herdman M, Stanier RY (1979). Generic assignments, strain histories and properties of pure cultures of cyanobacteria. Microbiology.

[33] Liu L, Jokela J, Wahlsten M, Nowruzi B, Permi P, Zhang YZ (2014). Nostosins, trypsin inhibitors isolated from the terrestrial cyanobacterium Nostoc sp. strain FSN. J Nat Prod.

[34] Sardari RR, Kulcinskaja E, Ron EY, Björnsdóttir S, Friðjónsson ÓH, Hreggviðsson GÓ (2017). Evaluation of the production of exopolysaccharides by two strains of the thermophilic bacterium Rhodothermus marinus. Carbohyd Polym.

[35] De Philippis R, Paperi R, Sili C, Vincenzini M (2003). Assessment of the metal removal capability of two capsulated cyanobacteria, Cyanospira capsulata and Nostoc PCC7936. J Appl Phycol.

[36] Micheletti E, Colica G, Viti C, Tamagnini P, De Philippis R (2008). Selectivity in the heavy metal removal by exopolysaccharide-producing cyanobacteria. J Appl Microbiol.

[37] Zinicovscaia I, Rudi L, Valuta A, Cepoi L, Vergel K, Frontasyeva MV (2016). Biochemical changes in Nostoc linckia associated with selenium nanoparticles biosynthesis. Ecol Chem Eng.

[38] Mota R, Rossi F, Andrenelli L, Pereira SB, De Philippis R, Tamagnini P (2016). Released polysaccharides (RPS) from Cyanothece sp CCY 0110 as biosorbent for heavy metals bioremediation: interactions between metals and RPS binding sites. Appl Microbial Biotechnol.

[39] Sampathkumar Y, Halith AM. GCMS Determination of Anticancer, Anti inflammatory and Anti bacterial compounds from salt tolerance Microalgae (Lyngbya sp. Nostoc sp. and Phormidium sp.) Isolated from Marakkanam Salt Pan, Tamil Nadu, India. 2020;11:1139-52.

[40] El Bestawy E (2019). Efficiency of immobilized cyanobacteria in heavy metals removal from industrial effluents. Desalination Water Treat.

[41] El-Sheekh MM, El-Shouny WA, Osman ME, El-Gammal EW (2005). Growth and heavy metals removal efficiency of Nostoc muscorum and Anabaena subcylindrica in sewage and industrial wastewater effluents. Environ Toxicol Pharmacol.

[42] Giner-Lamia J, Pereira SB, Bovea-Marco M, Futschik ME, Tamagnini P, Oliveira P (2016). Extracellular proteins: novel key components of metal resistance in cyanobacteria?. Front Microbiol.

[43] Bhunia B, Uday USP, Oinam G, Mondal A, Bandyopadhyay TK, Tiwari ON (2018). Characterization, genetic regulation and production of cyanobacterial exopolysaccharides and its applicability for heavy metal removal. Carbohyd Polym.

[44] Chug R, Mathur S (2013). Extracellular polymeric substances from cyanobacteria: characteristics, isolation and biotechnological applications-a review. Int J Adv Eng Sci Technol.

[45] Li P, Harding SE, Liu Z (2001). Cyanobacterial exopolysaccharides: their nature and potential biotechnological applications. Biotechnol Genet Eng Rev.

[46] Floutya R, El-Khourya J, Maatouka E, El-Samrania A (2019). Optimization of Cu and Pb biosorption by Aphanizomenon ovalisporum using Taguchi approach: kinetics and equilibrium modeling. Desalin Water Treat.

[47] Pereira S, Micheletti E, Zille A, Santos A, Moradas-Ferreira P, Tamagnini P (2011). Using extracellular polymeric substances (EPS)-producing cyanobacteria for the bioremediation of heavy metals: do cations compete for the EPS functional groups and also accumulate inside the cell?. Microbiology.

[48] Maalej H, Hmidet N, Boisset C, Buon L, Heyraud A, Nasri M (2015). Optimization of exopolysaccharide production from P seudomonas stutzeri AS 22 and examination of its metal-binding abilities. J Appl Microbiol.

[49] Yu H, Jia S, Dai Y (2010). Accumulation of exopolysaccharides in liquid suspension culture of Nostoc flagelliforme cells. Appl Biochem Biotechnol.

[50] Vonshak A, Cheung SM, Chen F (2000). Mixotrophic growth modifies the response of Spirulina (Arthrospira) platensis (Cyanobacteria) cells to light. J Phycol.

[51] Trabelsi L, Ouada HB, Bacha H, Ghoul M (2009). Combined effect of temperature and light intensity on growth and extracellular polymeric substance production by the cyanobacterium Arthrospira platensis. J Appl Phycol.

[52] Fábregas J, García D, Morales ED, Lamela T, Otero A (1999). Mixotrophic production of phycoerythrin and exopolysaccharide by the microalga Porphyridium cruentum. Cryptogamie Algologie.

[53] Iqbal M, Zafar S (1993). Effects of photon flux density, CO 2, aeration rate, and inoculum density on growth and extracellular polysaccharide production byPorphyridium cruentum. Folia Microbiol.

[54] Cheirsilp B, Mandik YI, Prasertsan P (2016). Evaluation of optimal conditions for cultivation of marine Chlorella sp as potential sources of lipids, exopolymeric substances and pigments. Aquac Int.

[55] Wu N, Li Y, Lan CQ (2011). Production and rheological studies of microalgal extracellular biopolymer from lactose using the green alga Neochloris oleoabundans. J Polym Environ.

[56] Tchounwou PB, Yedjou CG, Patlolla AK, Sutton DJ. Heavy metal toxicity and the environment. Molecular, clinical and environmental toxicology. 2012;5:133–64. 10.1007/978-3-7643-8340-4_6 PMC4144270 22945569

[57] Mohite BV, Koli SH, Narkhede CP, Patil SN, Patil SV (2017). Prospective of microbial exopolysaccharide for heavy metal exclusion. Appl Biochem Biotechnol.

[58] El-Enany A, Issa A (2000). Cyanobacteria as a biosorbent of heavy metals in sewage water. Environ Toxicol Pharmacol.

[59] Rossi F, De Philippis R (2015). Role of cyanobacterial exopolysaccharides in phototrophic biofilms and in complex microbial mats. Life.

[60] Chan S, Chow H, Wong MH (1991). Microalgae as bioabsorbents for treating mixture of electroplating and sewage effluent. Biomedical and environmental sciences: BES.

[61] Kazy SK, Sar P, Singh S, Sen AK, D’souza S. Extracellular polysaccharides of a copper-sensitive and a copper-resistant Pseudomonas aeruginosa strain: synthesis, chemical nature and copper binding. World J Microbiol Biotechnol. 2002;18(6):583–8.

[62] Ozturk S, Aslim B (2008). Relationship between chromium (VI) resistance and extracellular polymeric substances (EPS) concentration by some cyanobacterial isolates. Environ Sci Pollut Res.

[63] Gómez-Ordóñez E, Rupérez P (2011). FTIR-ATR spectroscopy as a tool for polysaccharide identification in edible brown and red seaweeds. Food Hydrocolloids.

[64] Leal D, Matsuhiro B, Rossi M, Caruso F (2008). FT-IR spectra of alginic acid block fractions in three species of brown seaweeds. Carbohyd Res.

[65] Shekhar SH, Lyons G, McRoberts C, McCall D, Carmichael E, Andrews F (2012). Brown seaweed species from Strangford Lough: compositional analyses of seaweed species and biostimulant formulations by rapid instrumental methods. J Appl Phycol.

[66] Ponnuswamy I, Madhavan S, Shabudeen S (2013). Isolation and characterization of 1663 green microalgae for carbon sequestration, waste water treatment and bio-fuel production. Bio-Sci Bio-Technol.

[67] Delattre C, Pierre G, Laroche C, Michaud P (2016). Production, extraction and characterization of microalgal and cyanobacterial exopolysaccharides. Biotechnol Adv.

[68] Shuhong Y, Meiping Z, Hong Y, Han W, Shan X, Yan L (2014). Biosorption of Cu2+, Pb2+ and Cr6+ by a novel exopolysaccharide from Arthrobacter ps-5. Carbohyd Polym.

[69] Agronematol EJ. Biocontrol of Root knot Nematode, Meloidogyne incognita infected banana plants by Cyanobacteria. 2013;12:113-29.

[70] Kanimozhi R, Prasath DA, Dhandapani R, Sigamani S (2021). In vitro antioxidant and antibiofilm activities of microcystis sp. against multidrug-resistant human pathogens. Ann Romanian Soc Cell Biol.

[71] Wu J-T, Chiang Y-R, Huang W-Y, Jane W-N (2006). Cytotoxic effects of free fatty acids on phytoplankton algae and cyanobacteria. Aquat Toxicol.

[72] Mundt S, Kreitlow S, Jansen R (2003). Fatty acids with antibacterial activity from the cyanobacterium Oscillatoria redekei HUB 051. J Appl Phycol.

[73] Alghamdi S, Migdadi H, Khan M, El-Harty EH, Ammar M, Farooq M, et al. Phytochemical profiling of soybean (Glycine max (L.) Merr.) genotypes using GC-MS analysis. Phytochemicals-Source of Antioxidants and Role in Disease Prevention. 2018;1:139-41. 10.1016/j.sjbs.2017.10.014 PMC5775105 29379350

[74] Keskın D, Ceyhan N, Uğur A, Dbeys AD (2012). Antimicrobial activity and chemical constitutions of West Anatolian olive (Olea europaea L.) leaves. J Food Agric Environ.

